# Antibiotic Prophylaxis in Patients Undergoing Oncologic Head and Neck Surgery with Free Flap Reconstruction: Still a Matter of Debate

**DOI:** 10.3390/antibiotics14111160

**Published:** 2025-11-15

**Authors:** Femke Goormans, Auke van Mierlo, Isabel Spriet, Gaétan Van de Vyvere, Bart Knockaert, Robin Willaert

**Affiliations:** 1 Department of Oral and Maxillofacial Surgery, Faculty of Medicine KU Leuven, University Hospitals Leuven, Campus Sint-Rafaël, Kapucijnenvoer 7, 3000 Leuven, Belgium; 2 Pharmacy Department, Faculty of Medicine KU Leuven, University Hospitals Leuven, Herestraat 49, 3000 Leuven, Belgium; 3 Department of Pharmaceutical and Pharmacological Sciences, Clinical Pharmacology and Pharmacotherapy, Faculty of Medicine KU Leuven, Campus Gasthuisberg, Herestraat 49, 3000 Leuven, Belgium; 4 Department of Oral and Maxillofacial Surgery, AZORG Ziekenhuizen, Moorselbaan 164, 9300 Aalst, Belgium; 5 OMFS-IMPATH Research Group, Department of Imaging and Pathology, Faculty of Medicine KU Leuven, University Hospitals Leuven, Campus Sint-Rafaël, Kapucijnenvoer 7, 3000 Leuven, Belgium

**Keywords:** head and neck surgery, surgical site infections, perioperative antibiotic prophylaxis, free flap reconstruction, infection prevention, antimicrobial resistance, antimicrobial stewardship

## Abstract

**Background/Objectives**: Surgical site infection (SSI) significantly impacts patient outcomes in oncologic head and neck surgery with free flap reconstruction. Perioperative antibiotic prophylaxis (PAP) is widely accepted to prevent SSI. Despite decades of research, infection rates often exceed 40%, and controversies remain regarding antibiotic type and duration. While the literature on general head and neck surgery is abundant, it does not fully address the unique challenges of oncologic patients undergoing complex free flap reconstruction in the head and neck region. This review assesses the evidence for PAP in this population and examines concerns related to antimicrobial resistance (AMR). **Methods**: We conducted a review of clinical trials, systematic reviews, and relevant literature on PAP in oncologic head and neck surgery with free flap reconstruction. Key aspects included antibiotic type, timing, duration, and impact on SSI rates and patient outcomes. General head and neck surgery literature was considered when procedure-specific data were lacking. **Results**: PAP reduces SSI rates, but clinical practice varies regarding antibiotic choice and duration. Short-term prophylaxis may suffice for some procedures, whereas prolonged regimens are often used despite limited additional benefit. A multidisciplinary approach considering procedure-specific risks and patient factors improves outcomes. The risk of AMR underscores the need for standardized, evidence-based protocols. Significant gaps remain, particularly concerning optimal PAP regimens for free flap reconstruction. **Conclusions**: PAP is essential for SSI prevention in head and neck oncologic surgery with free flap reconstruction, yet current practices are heterogeneous. Standardized, procedure-specific protocols are needed to optimize prophylaxis, reduce SSI rates, and limit AMR, ultimately improving patient care and outcomes.

## 1. Introduction

Oncologic head and neck surgery with free flap reconstruction has evolved into a highly complex domain of surgical practice. This complexity arises from the nature of these procedures, which often involve an extended duration of surgery, the use of sophisticated (often patient specific) osteosynthetic material, the presence of a tracheostomy, and oropharyngeal microorganisms at the surgical site. Additionally, a vulnerable patient population—characterized by factors such as comorbidities, preoperative smoking, and previously irradiated tissue—further contributes to the elevated risk of surgical site infection (SSI) [1,2,3,4]. SSI can lead to significant morbidity, prolonged hospital stays, and increased healthcare costs [2,5].

Perioperative antibiotic prophylaxis (PAP) has been widely accepted as a primary strategy to prevent SSI in this specific patient population [6]. However, despite decades of dedicated research [7,8,9,10,11,12], reported SSI rates continue to exceed 40% [7,13,14]. Surprisingly, there has been little progress in the development of effective prevention and treatment strategies. Therefore, controversies persist regarding the type and duration of PAP, not only in the literature but also in our daily clinical practice where antibiotics are often continued for 7 days postoperatively or longer. Our institution, a tertiary referral center annually treating more than 50 patients with head and neck tumor resections and free flap reconstructions, reflects the practice in a high-volume setting. This underscores the pressing need for a reevaluation of our current practice. The increasing concern of antimicrobial resistance (AMR) further amplifies the importance of reevaluating prophylactic strategies, urging a rational use of antibiotics to prevent the emergence of drug-resistant strains [15].

One of the critical challenges is our limited understanding of the causative microorganisms responsible for SSI in this specific surgical context. Current protocols for obtaining cultures from infected surgical sites have not been uniformly implemented or standardized, leading to a lack of precise data on the local microbial landscape. This lack of data compromises our ability to tailor antimicrobial prophylaxis effectively. Furthermore, a clear and context-specific definition for SSI following head and neck surgery is lacking [16].

In light of these persistent challenges and knowledge gaps, the purpose of this narrative review is to thoroughly evaluate the present state of PAP in oncologic head and neck surgery with free flap reconstruction. A systematic review with meta-analysis was not feasible due to significant heterogeneity across studies, including variability in patient populations, surgical techniques, antibiotic regimens, and definitions of SSI. Moreover, most existing systematic reviews provide broad overviews of antibiotic prophylaxis for general head and neck surgery, without addressing the need for tailored protocols specific to free flap reconstruction or the oncologic patient. Given that infection—the primary outcome measure—is often poorly defined or inconsistently reported, comparing studies remains problematic and hampers evidence synthesis.

To identify relevant studies, we consulted several databases, including PubMed, Embase, and Cochrane Library. Our search strategy was conducted with a timeframe of publications from the past 20 years (2005–2025) and focused on studies related to PAP in head and neck oncology with a particular emphasis on free flap reconstruction. By synthesizing existing research and acknowledging diverging hypotheses, we aim to provide a comprehensive overview of the field’s advancements, challenges, and potential future directions. In doing so, this review intends to contribute to the ongoing debate surrounding infection prevention strategies, while highlighting the principal conclusions drawn from the literature.

## 2. The Rationale for Antibiotic Prophylaxis in Head and Neck Surgery

In head and neck surgery with free flap reconstruction, prevention of SSI is a major concern due to its potential to compromise both patient outcome and quality of life [2,5]. The clinical relevance of SSI in head and neck surgery with free flap reconstruction is clear when we look at epidemiological data. Studies have shown SSI rates ranging from 10% to 46% [7,13,14]. SSI rates vary widely based on factors including the type of procedure, patient characteristics, and surgical technique. These SSIs lead to longer hospital stays, often necessitate additional treatment, and can negatively affect both functional and aesthetic outcome [14,17]. In some cases, they may also affect prognosis by delaying the initiation of adjuvant therapy. The financial costs related to SSI are significant [18], emphasizing the need to urgently create and implement effective preventive strategies. PAP is such a strategy, and scientific data already proved its positive impact in general head and neck surgery [19,20,21,22,23,24,25]. However, the issue is that this specific patient population has many intrinsic risk factors that increase the overall SSI risk, which could validate a tailored approach to PAP.

### 2.1. Procedure-Specific Risk Factors for SSI

**Figure 1:**
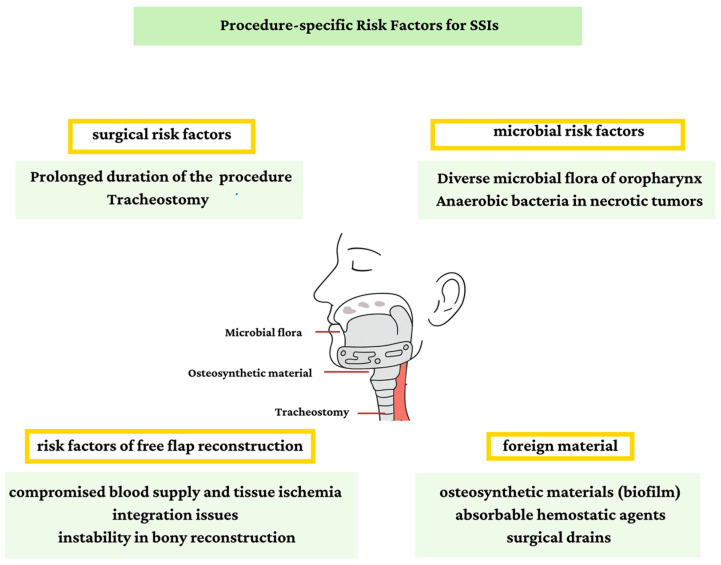
Procedure-specific risk factors for SSI.

There are several risk factors that contribute to the increased risk of SSI in these procedures (Figure 1). Prolonged duration of the surgical procedure, often exceeding 8 h in complex head and neck reconstructions, exposes patients to an increased risk for microbial contamination [3]. Additionally, the oral and pharyngeal regions harbor a diverse microbial flora, making these sites susceptible to colonization [26,27]. Importantly, head and neck oncological surgery almost invariably combines a non-sterile intraoral approach with an initially sterile cervical approach, often resulting in continuity between both surgical fields, which further amplifies the risk of contamination. In necrotic or ulcerated tumors, there is an increased colonization by anaerobic bacteria [28]. The necessity for a tracheostomy further increases the infection risk due to the continuous communication with the respiratory tract [29]. Additionally, foreign materials such as absorbable hemostatic agents or surgical drains harbor an additional infection risk [14,30].

In the context of free flap reconstruction, a conductive environment for bacterial growth is established due to the compromised blood supply to the reconstructed flap, tissue ischemia, and possible integration issues [31,32]. Moreover, it is imperative to underscore the critical distinction between osseous (bony) free-flap reconstruction and soft tissue free-flap reconstruction. An osseous free-flap reconstruction harbors a higher infection risk, given that bony instability results in impaired neovascularity, ongoing soft-tissue trauma and osteolysis, which in turn promote bacterial proliferation [33]. Furthermore, patient-specific osteosynthetic materials introduce an additional infection risk as these materials can serve as potential sites for bacterial adherence and biofilm formation. Therefore, it is of paramount importance to consider the type of reconstruction (i.e., bony involvement) when formulating procedure-specific PAP protocols.

### 2.2. Patient-Specific Risk Factors for SSI

**Figure 2:**
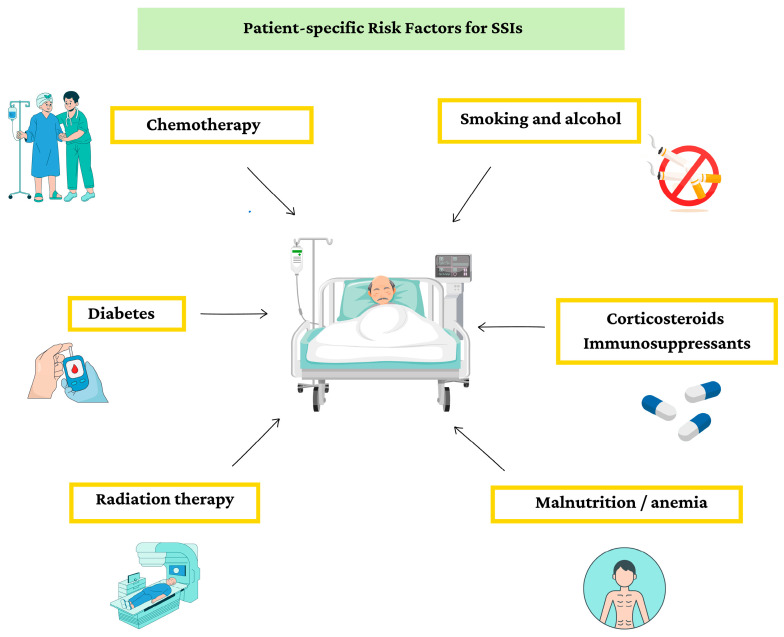
Patient-specific risk factors for SSI.

Oncologic patients face an elevated SSI risk due to a combination of factors associated with their disease and treatment (Figure 2). Firstly, tumors often weaken the immune system by producing immunosuppressive factors and by placing a considerable metabolic burden on the body, thereby impairing its ability to mount a robust defense against potential pathogens [4]. Secondly, treatments such as radiation therapy and Systemic Anti-Cancer Therapy (SACT) can further suppress immune function [4]. Cytotoxic SACT (i.e., chemotherapy) can lead to temporary neutropenia, a condition characterized by a decreased number of infection-fighting white blood cells, making patients more vulnerable to bacterial invasion [29,34]. However, in our patient population, chemotherapy usually plays only a minor role in perioperative risk, since it is most often administered in the adjuvant setting after surgery. In contrast, radiation therapy can have lasting effects by impairing tissue vascularity and wound healing, creating an environment prone to infections [35].

Furthermore, specific risk factors can further amplify the susceptibility of oncologic patients to SSI. Lifestyle choices such as smoking, and alcohol abuse impair immune function and delay wound healing [36]. Malnutrition and anemia, common in oncologic patients, compromise the body’s ability to mount an effective immune response and impede tissue repair [37,38]. Conditions like diabetes, especially when poorly controlled, disrupt normal wound healing processes [39]. Finally, pre-existing immunosuppression, whether caused by underlying medical conditions or medications such as corticosteroids, further weakens the body’s defenses against pathogens [38]. Collectively, these specific risk factors compound the already heightened infection risk of oncologic patients, underscoring the critical importance of tailored perioperative care strategies to mitigate the risk of SSI in this patient population [29,39,40,41].

## 3. The Efficacy of Antibiotic Prophylaxis and Patient Outcome

The use of PAP in general head and neck surgery is supported by a number of studies that have investigated its impact on the prevention of SSI [19,20,21,22,23,24,25]. These studies consistently demonstrate a significant reduction in the overall SSI rate among patients who received PAP compared to those who did not. Consequently, a notable decrease is observed in complications related to SSI, including flap failure [7]. However, it is often difficult to determine causality in such cases—whether the infection led to flap failure or whether undetected early flap failure created conditions conducive to infection. In our own cohort, flap failure occurs in fewer than 5% of patients, whereas SSI rates are considerably higher. This indicates that infections frequently develop despite adequate flap viability, and that most flaps can be salvaged once the SSI is adequately controlled.

The observed reduction in SSI with PAP consistently translated into improved postoperative outcomes such as shorter hospital stays, a decreased need for secondary interventions and enhanced functional and aesthetic outcomes. These benefits contribute to improved patient well-being as well as the efficiency and cost-effectiveness of healthcare systems [5,18,42,43].

Despite these advantages, PAP should not be viewed as a standalone solution for preventing SSI. Effective SSI prevention also depends on rigorous perioperative hygiene and sterility, high-quality postoperative wound care, optimal preoperative management of comorbidities such as diabetes, smoking cessation, and minimizing surgical duration. PAP cannot compensate for shortcomings in these critical areas.

## 4. Antibiotic Type and Timing of Antibiotic Administration

Effective PAP relies on the optimal choice of antibiotic type, the appropriate route of administration (oral vs. intravenous (IV)), and precise timing of administration. In this subsection we discuss the rationale behind antibiotic choice, commonly used antibiotic agents and the diverse microorganisms encountered at the surgical site. Finally, we explore the critical aspect of timing of antibiotic administration in relation to the surgical incision to maximize its preventative impact.

### 4.1. Antibiotic Type and the Microbial Spectrum

The selection of antibiotic type should consider the susceptibility of the microorganisms commonly present in the head and neck region [44]. The predominant oropharyngeal organisms include both aerobic and anaerobic *Streptococcal species*, *Bacteroides species*, *Peptostreptococcus species*, *Enterobacterales*, and *Staphylococcal species* [1,45]. Their exact role in SSI is difficult to determine due to heterogeneous sampling and culture methods [1,46], and most microbiological data originate from non-surgical infections such as acute upper respiratory tract infections (URTI) or chronic sinusitis [1]. Data on pathogens identified through culture at time of SSI diagnosis are scarce. Only one study reported culture results from six deep abscesses after free flap reconstruction, which all showed the presence of *Streptococcus anginosus* [47]. Retrospective analyses of general head and neck cancer surgery suggest that when SSIs occur, they are typically polymicrobial, most often involving *Staphylococcus aureus* (including Methicillin-Resistant *Staphylococcus aureus* (MRSA)) (15%), and Gram-negative bacilli such as *Klebsiella species* (24%) and *Acinetobacter species* (15%) [48]. Reported multidrug resistance (MDR) in these settings is mainly related to MRSA and MDR Gram-negative bacilli, including Extended-spectrum beta-lactamase-producing *Enterobacterales* (ESBL-E) [48].

Future studies should aim to characterize the microbiology of SSI more accurately by using standardized and high-quality sampling methods. To ensure accurate culture results, high quality samples from deep tissues and osteosynthetic material specimens (i.e., sonication) are essential [49]. Ideally, microbial therapy is discontinued two weeks prior to sampling to avoid false-negative culture results [50]. Culture sampling should include aerobic and anaerobic organisms and susceptibility testing for β-lactams, clindamycin, and—depending on local epidemiology—MRSA and ESBL-E, to enable targeted antimicrobial therapy [48].

When pathogens originate primarily from skin flora, cefazolin is considered the preferred antibiotic due to its effectiveness against Gram-positive cocci (i.e., *Staphylococcal species*) [44]. However, head and neck procedures frequently expose the surgical field to oropharyngeal flora, necessitating broader-spectrum coverage (e.g., ampicillin-sulbactam, cefazolin with metronidazole) [51]. Allergies to penicillins or other betalactams necessitate alternative drug choices such as clindamycin or levofloxacin with metronidazole [44].

The classification of surgical wounds is relevant when selecting PAP in head and neck surgery but is often inconsistently applied in the literature [12,26]. According to the CDC guidelines, surgical wounds are classified as clean (class I), clean-contaminated (class II), contaminated (class III), or dirty-infected (class IV) [51]. In this classification, clean-contaminated wounds (class II) include procedures in which the oropharynx or upper aerodigestive tract is entered, thereby exposing the surgical field to oropharyngeal flora and making it inherently non-sterile. Contaminated wounds (class III), by contrast, are accidental wounds or procedures involving major breaks in sterile technique or significant spillage from the gastrointestinal tract [51]. Despite these standardized definitions, several studies in head and neck reconstructive surgery classify any involvement of the respiratory tract as contaminated (class III) rather than clean-contaminated (class II), leading to inconsistencies in terminology. This lack of clarity complicates interpretation and comparison of study outcomes, including assessments of the effectiveness and optimal duration of PAP. In this manuscript, we consistently use the term clean-contaminated to refer specifically to procedures where the surgical field becomes exposed to oropharyngeal flora.

### 4.2. Controversies on Antibiotic Choice

Available clinical trials show inconsistency in antibiotic type, with varying activity against the presumed bacterial flora. This poses a significant limitation, as using an effective antibiotic agent is crucial for validating study outcomes [16].

Mitchell et al. reported that antibiotic type appears to affect the rate of all SSIs more than prophylaxis duration, recommending ampicillin-sulbactam for clean-contaminated head and neck procedures [7]. Iocca et al. found comparable results for penicillins and cephalosporins; however, their study focused solely on head and neck cancer surgery and not specifically on free flap reconstruction [52]. Van Mierlo et al. illustrated a higher incidence of abscesses with cefazolin plus metronidazole in comparison to amoxicillin-clavulanate in a retrospective cohort study of 342 cases [47]. Several authors have shown increased infection rates with clindamycin [9,10,12,53,54], although Johnson et al. reported similar efficacy between cefazolin and clindamycin (600 mg) [55]. Use of clindamycin in patients with penicillin allergy is increasingly questioned due to rising resistance among *Staphylococcus species* [56], and studies confirm higher SSI rates with clindamycin compared to β-lactams [9,10,12,53,54]. Consequently, some centers use fluoroquinolone-based combinations (e.g., levofloxacin with metronidazole) for confirmed severe β-lactam allergy [56].

The existing research presents widely disparate conclusions, resulting in confusion and a lack of clear guidelines for clinical decision-making. However, establishing standardized studies is challenged by the lack of a definition of SSI, and the lack of cultures indicating the susceptibility of causative organisms. The IDSA guidelines by Bratzler et al. offer general clinical practice recommendations for antimicrobial prophylaxis in surgery; however, these are not tailored to oncologic reconstructions in the head and neck region involving free flaps [57]. In Table 1, we combine these guidelines with findings from relevant systematic reviews to propose more context-specific recommendations [10,58,59,60]. This should be interpreted with caution due to the limited and heterogeneous evidence available in this highly complex surgical context and vulnerable patient population.

### 4.3. Timing of Antibiotic Administration

The timing of antibiotic administration in relation to the surgical incision is critical for prophylactic efficacy. Administration 30–60 min prior to incision ensures optimal tissue concentrations during surgery, reducing the risk of infection with skin flora [61]. Redosing depends on the pharmacokinetic profile of each antibiotic (e.g., time to peak concentration in the tissue, and duration of action), and should also be considered in cases of major blood loss or transfusion [62].

As summarized in Table 1, the American Society of Health-System Pharmacists (ASHP) recommends ampicillin-sulbactam 3 g IV (2 g ampicillin/1 g sulbactam) as first-line prophylaxis for average weight adults with normal renal function in the United States (US) [57]. In the European Union (EU), where ampicillin-sulbactam is unavailable, amoxicillin-clavulanic acid 1 g IV is the preferred equivalent first-line option [59,60]. Cefazolin 2 g IV, when combined with metronidazole 1.5 g IV (or ornidazole 1 g IV in the EU), serves as an alternative regimen in both the US and EU [57,59,60]. Intraoperative redosing should occur if the procedure exceeds 3 h or if there is significant blood loss. In patients with a confirmed β-lactam allergy, levofloxacin combined with metronidazole is recommended [58]. Clindamycin is less preferred due to higher SSI rates [56] (Table 1).

**Table 1:** Recommended Doses and Redosing Intervals for Antibiotic Prophylaxis in Oncologic Head and Neck Surgery with Free flap reconstruction [10,47,57,58,59,60].

Indication	Antibiotic Type and Dosing (United States) [57]	Antibiotic Type and Dosing (European Union) [59,60]	Intraoperative Redosing [57,59,60] ^1^	Recommended Postoperative Redosing Interval [57,59,60] ^1^	Duration of Postoperative Antibiotic Prophylaxis ^2^
**Oncological head and neck surgery with free flap** **reconstruction**	**Ampicillin** 2 g–**Sulbactam** 1 g IV *(children 50 mg/kg BW IV)*	**Amoxicillin** 1 g—**Clavulanic Acid** 200 mg IV *(children 25 mg/kg BW IV)*	US 2 h EU 3 h–7 h–11 h	4 h	**Soft tissue reconstruction:** **24–48 h ^2^** **Bony reconstruction:** **72 h ^2^**
**Alternative** **regimen**	**Cefazolin** 2 g IV (>120 kg Cefazolin 3 g IV) *(children 30 mg/kg BW IV)* + **Metronidazole** 500 mg IV *(children 20 mg/kg BW IV)*	**Cefazolin** 2 g IV (>120 kg Cefazolin 3 g IV) *(children 30 mg/kg BW IV)* + **Ornidazole** 1 g IV or **Metronidazole** 500 mg IV *(children 20 mg/kg BW IV)*	US 4 h EU 3 h–8 h–16 h 24 h	8 h 24 h	
**Confirmed** **β-lactam allergy**	**Levofloxacin** 500 mg IV or **Clindamycin** 900 mg IV ^3^ *(children 10 mg/kg BW IV)* + **Metronidazole** 500 mg IV *(children 15 mg/kg BW IV)*	**Levofloxacin** 500 mg IV or **Clindamycin** 600 mg IV ^3^ *(children 10 mg/kg BW IV)* *+* **Ornidazole** 1 g IV or **Metronidazole** 500 mg IV *(children 15 mg/kg BW IV)*	12 h US 6 h, EU 8 h 24 h	12 h US 6 h, EU 8 h 24 h	

^1^ These recommendations apply to current clinical guidelines used in both the United States and the European Union regarding antibiotic prophylaxis [51,57,59,60]. ^2^ Preliminary proposal of prophylaxis duration depending on reconstruction type: 24–48 h for soft-tissue reconstruction; 72 h for bony reconstruction involving osteosynthesis. ^3^ Clindamycin is associated with higher SSI rates and rising Staphylococcus resistance; levofloxacin is often used as an alternative [56]. (Abbreviations: BW, bodyweight; IV, intravenous; h, hours).

### 4.4. Controversies on Extended Prophylaxis Duration

The optimal duration of PAP is still up for debate. Shorter courses are favored to minimize the risk of side effects and AMR, and the majority of studies show no benefit of prolonging PAP over 24 h [7,10,11,43,63,64] or 48 h [8,12,52,65], postoperatively. These findings align with expectations, given that PAP theoretically prevents SSI caused by perioperative contamination. We found only one study that showed a significant decrease in SSI rates when antibiotics were continued for up to 5 days postoperatively. However, it is worth noting that in the comparison group, antibiotics were stopped right after the surgery was completed, rather than continued for the usual 24 h postoperative period [66].

However, a point of concern is head and neck oncologic surgery involving bony reconstruction. In these procedures, osteosynthetic materials and implants are introduced into clean-contaminated environments—a practice that would be unacceptable in orthopedic trauma surgery, where strict sterility is essential. In the oral cavity, a microbiologically dense site, this creates a high risk of early bacterial colonization and biofilm formation on the implanted hardware, which is difficult to eradicate and can lead to persistent infections and implant failure. Moreover, the biomechanical instability that often accompanies bony reconstruction further increases susceptibility to infection [67]. Even limited interventions—such as mandibular rim resections—involve hardware and bone exposure, prompting consideration of extended prophylaxis. In these contexts, prolonging PAP over 48 h is commonly practiced to reduce the risk of early microbial colonization [68], although no direct evidence exists to support its efficacy specifically in head and neck oncologic reconstruction.

In current clinical practice, antibiotics are frequently continued for 7 days postoperatively, based on the rationale that extended use might prevent SSI. However, the literature does not support this approach, making its effectiveness unclear and highlighting the need to explore alternative preventive strategies. The practice of prolonging antibiotics up to 72 h in cases involving bony reconstruction likely stems from musculoskeletal trauma surgery protocols for open fractures [68]. Yet, extending PAP beyond this period lacks evidence, underscoring the need for more targeted research and evidence-based guidelines.

Given the lack of clear evidence and the heterogeneity of surgical scenarios, a “one-size-fits-all” strategy for PAP duration does not appear appropriate in head and neck oncologic surgery with free flap reconstruction. Therefore, we propose a procedure-specific approach. In cases involving soft-tissue reconstruction only, PAP should be limited to 24–48 h. In contrast, when bony reconstruction is performed—particularly in clean-contaminated fields where osteosynthetic material is exposed to oral or oropharyngeal flora—we propose extending PAP to 72 h, as these procedures carry a higher risk of early bacterial colonization and biofilm formation on the hardware (Table 1). Importantly, this represents a preliminary proposal. Validation through future studies with robust methodology, standardized outcome measures, and procedure-specific subgroup analysis is essential. Such research will be necessary to refine this approach and ultimately establish evidence-based guidelines that can reliably support clinical decision-making in this highly specialized surgical context.

## 5. Implications of Antibiotic Overuse

### 5.1. The Challenge of AMR

The overuse and misuse of antibiotics are considered significant contributing factors to the rise and spread of AMR, compromising the efficacy of antibiotic agents, and limiting treatment options for infections [15,69,70,71]. AMR occurs as an innate evolutionary reaction to exposure to antimicrobial agents, wherein microorganisms acquire the capacity to endure these agents through genetic alterations within chromosomal genes and via horizontal gene transfer [71]. The worldwide spread of AMR has the potential to jeopardize our capacity to combat prevalent and emerging infectious diseases, while also undermining various other advancements in healthcare. It is imperative for head and neck surgeons, along with all healthcare professionals, to acknowledge AMR as a developing global health crisis.

Antimicrobial stewardship is an important strategy to combat AMR [72,73]. Based on evidence-based practices that aim to optimize antibiotic use, unnecessary administration of antibiotics is reduced, and the emergence of resistant pathogens minimized [72]. Incorporating these principles in the context of antibiotic prophylaxis involves careful evaluation of the risks and benefits of administering antibiotics, and responsible use of antimicrobial agents based on available guidelines.

### 5.2. Adverse Effects of Antibiotic Overuse

Apart from fueling AMR, antibiotic overuse can lead to various adverse effects. Prolonged or unnecessary antibiotic courses disrupt the microbiome, potentially causing diarrhea, nutrient imbalances, and weakened immunity [74,75]. Additionally, extended antibiotic use can make patients more susceptible to opportunistic infections such as Candida Albicans [74]. Allergic reactions, from mild rashes to severe anaphylaxis, pose immediate risks [76]. Some studies suggest long-term health implications, including metabolic disturbances and an increased risk of metabolic and cardiovascular disease [77]. Furthermore, antibiotics released into the environment can contribute to ecological imbalances, impacting both human and animal health [78]. To optimize patient outcomes and preserve antibiotic efficacy, limiting the duration of antibiotic use is crucial while considering these side effects.

### 5.3. The Impact of Antibiotic Therapy on Cancer Therapy Outcomes

While antibiotics have proven beneficial in preventing and managing SSI in oncologic patients, a growing body of evidence suggests their potential negative impact on anticancer treatment effectiveness and cancer-specific survival, particularly in patients receiving immunotherapy [38,79,80]. It is now recognized that the microbiome plays a role in modifying antitumor immunity and influencing the effectiveness of cancer therapies through systemic immune responses [79]. Given these insights, it is advisable to minimize the use of broad-spectrum antibiotics in patients treated with immunotherapy whenever feasible [81]. Additionally, beyond the type of antibiotic, the duration of treatment seems critical, with the harmful effects most commonly observed in patients undergoing extended or repeated cycles of antibiotic therapy [82,83].

Moreover, administering antibiotics during curative chemoradiotherapy for locally advanced head and neck cancers has been linked to a notable decrease in progression-free, overall, and disease-specific survival rates [84]. Disruption of the oral and gut microbiota may impair treatment response and promote locoregional relapse. Consequently, the potential risks associated with broad-spectrum and prophylactic antibiotic therapy in these patients should be thoroughly considered.

## 6. Alternative Approaches and Recent Advancements in the Field of Infection Prevention

Infection prevention involves a multi-faceted approach, encompassing alternative prevention strategies and novel techniques that extend beyond systemic antibiotics.

### 6.1. Preoperative Screening and Patient Optimization

Managing comorbidities and evaluation of liver, kidney and thyroid function are paramount in a preoperative patient assessment [40,85]. Special attention should be given to the elimination of oral foci, both dental and periodontal, to optimize the oral microbiota before initiating any form of therapy [86]. Encouraging patients to quit smoking prior to surgery can further reduce the risk of postoperative complications, including SSI [40,85]. In addition, preoperative screening for multidrug-resistant organisms, such as MRSA, is advisable. When detected, targeted decolonization can be initiated prior to surgery to further reduce the risk of SSI.

### 6.2. Perioperative Antisepsis

Skin antisepsis with agents such as chlorhexidine or iodine reduces the microbial load on the surgical site and lowers infection risks [40,51]. In head and neck surgery, additional measures include antimicrobial mouthwash prior to surgical incision and intraoperative irrigation of the surgical field. Selective oropharyngeal decontamination (SOD), typically using chlorhexidine, has shown to reduce the incidence ventilator-associated pneumonia and other infections in the ICU [87]. Strict adherence to aseptic techniques and maintaining a sterile environment during surgery is paramount [40,51]. Finally, minimizing the duration of surgical site exposure reduces infection risks [3].

### 6.3. Wound Drains and Coverings

Closed suction drains (CSD) can help remove excess fluid and reduce the risk of hematoma and SSI [88]. However, prolonged use may increase the infection risk. Drains are typically removed on the third postoperative day or when the output falls below 30 cubic centimeters in a 24 h span, although recent evidence shows no clear link between drain duration or output and wound complications [88], indicating the need for further research. Appropriate wound care, using specialized dressings or wound coverings with antimicrobial properties can further help prevent SSI.

### 6.4. Patient Education and Regular Follow-Up

Providing patients with clear instructions on wound care, early infection signs and indications to seek medical attention can empower them to actively participate in infection prevention. Furthermore, regular, and long-term medical follow-up allows for early detection and intervention.

### 6.5. Novel Approaches to Infection Prevention

In response to the growing concern of AMR, innovative infection prevention methods are becoming more important. The goal of these strategies is to target microorganisms at their root, offering an alternative to conventional systemic antibiotic-centered approaches.

#### 6.5.1. Local Antibiotic Prophylaxis

Topical or local antibiotic administration has long been used to prevent SSI [89]. In general and orthopedic trauma surgery, antibiotics can be delivered directly into the surgical field via carriers that release the drug over several days, also helping to manage dead space [89,90]. To date, this approach has not been described for head and neck surgery with free flap reconstruction. The supplementary use of local antimicrobial substances can enhance prophylaxis effectiveness beyond what can be attained through systemic administration alone. High antimicrobial concentrations can be achieved at the surgical site—even in areas with limited vascularity—while reducing systemic exposure and potential toxicity [90].

#### 6.5.2. Immunomodulatory Agents

Immunomodulatory prophylaxis aims to strengthen the host immune response rather than act directly on bacteria [91]. Agents such as cytokines, antimicrobial peptides, or Toll-like receptor agonists may enhance local immune activation and reduce SSI [92], particularly in immunocompromised patients. These therapies could potentially be combined with antibiotics and are less likely to induce dysbiosis or resistance [91]. However, this approach is not currently used in clinical practice and remains an area of active research.

#### 6.5.3. Biofilm Disruption Agents

Microbial adhesion to osteosynthetic material often leads to biofilm formation, which is a major cause of persistent infections following bony reconstruction [93]. The pathogens most frequently involved include *Pseudomonas aeruginosa*, *Escherichia coli*, and *Staphylococcus aureus* [94]. IV antibiotic therapy alone is rarely successful in treating biofilm infections and subtherapeutic exposure may promote AMR [95,96]. Promising approaches include anti-biofilm surface coatings and agents that inhibit early biofilm formation, such as 2-aminoimidazole–based compounds, which have shown activity against Staphylococcus aureus in experimental models for orthopedic device-related infections [93,97]. Preventive measures should focus on minimizing implant contamination and improving implant surface design to reduce bacterial adhesion.

## 7. Limitations of Existing Evidence

Evaluating PAP in this specific surgical field is difficult due significant limitations in the available evidence. Studies vary widely in antibiotic type and duration, surgical techniques, outcome measures, and anatomical sites, making comparisons and guideline development challenging. The overall methodological quality is low, and there is a lack of high-quality, procedure-specific randomized controlled trials (RCTs), which limits the strength of conclusions. As a result, determining the most effective and optimal PAP protocol remains challenging.

Furthermore, the criteria used to measure outcomes in these studies are frequently unclear. More specifically, a standardized definition of SSI in the field of head and neck surgery is nonexistent [16]. Although the CDC criteria for SSI are often used, they are not specific to this patient population. Many studies do not clearly define SSI criteria and follow-up periods are often insufficient to capture all infections [16]. Additionally, culture results are frequently unavailable at the time of infection diagnosis. This makes it difficult to develop PAP protocols based on causative microorganisms. These inconsistencies make synthesis of the evidence difficult and emphasize the need for better-designed studies with clear outcome measures.

A final limitation is that most studies do not distinguish between patients with and without bony reconstruction, a clinically relevant difference. Bony reconstruction involves osteosynthetic material and biomechanical instability, both known to increase infection risk [33,67].

## 8. Future Directions and Recommendations

Given the limited and heterogeneous evidence currently available, future research should focus on developing evidence-based, procedure-specific PAP protocols. High-quality methodology and standardized outcome measures are essential. A consistent definition of SSI in head and neck surgery is needed, distinguishing between donor- and recipient-site infections, incorporating culture results, and applying standardized follow-up to improve comparability between studies. A key priority is research on cases requiring osteosynthetic materials, as these carry a higher infection risk due to early biofilm formation and mechanical instability.

**Figure 3:**
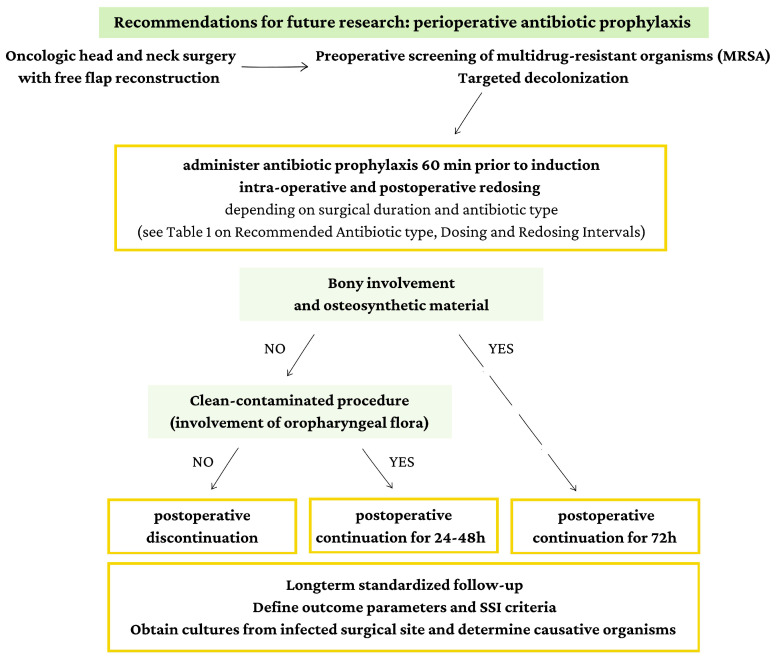
Recommendations for future research and preliminary proposal for tailored PAP duration based on surgery type.

Large-scale comparative studies and RCTs are needed to validate this tailored approach. To guide future research, we propose a procedure-specific approach to PAP based on the involvement of bone in the surgical field and whether it is considered clean-contaminated (involving oropharyngeal flora). Figure 3 outlines a possible guideline that accounts for these critical factors, providing a structured framework for implementing PAP protocols in head and neck surgery with free flap reconstruction. This figure represents a preliminary proposal, and further research is imperative to validate and refine this approach. Ongoing studies with robust methodologies and comprehensive outcome assessments will be crucial in establishing evidence-based guidelines that can support clinical decision-making in this specialized surgical context.

**Figure 4:**
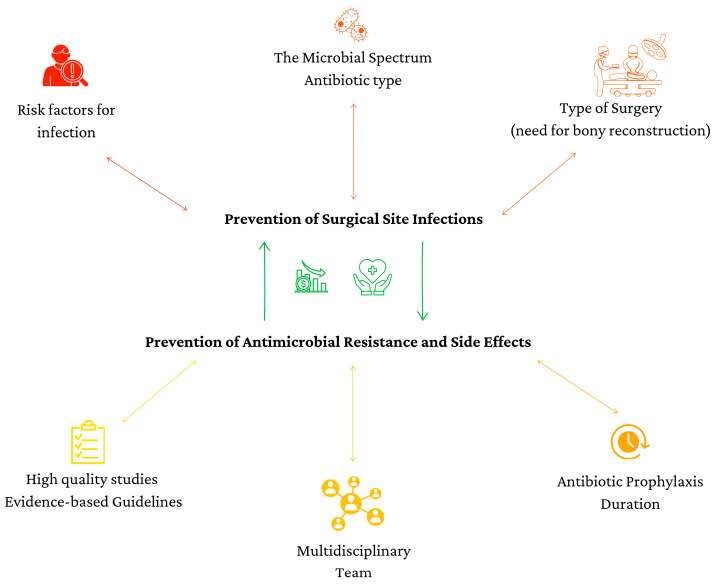
Key factors for improving infection prevention and AMR.

Beyond PAP duration and antibiotic selection, infection prevention also depends on broader perioperative decision-making. Figure 4 highlights additional factors that should guide PAP decisions, including microbial spectrum, patient-specific risk factors, surgical complexity, and multidisciplinary collaboration. Considering these factors will support rational antibiotic use, improve infection prevention, and help mitigate AMR.

## 9. Conclusions

Oncologic head and neck surgery with free flap reconstruction has a high risk of SSI. Despite decades of research and a wide acceptance of PAP as a primary preventive strategy, persistently high infection rates remain a significant concern. This review highlights ongoing uncertainty regarding the optimal antibiotic choice and duration, driven by heterogeneous evidence and the lack of a clear SSI definition specific to this field.

While studies show varied outcomes, ampicillin-sulbactam or amoxicillin-clavulanic acid are generally preferred. Current evidence supports limiting PAP to 24–48 h in soft-tissue reconstruction, whereas up to 72 h of PAP appears justified in bony reconstruction. Prolongation beyond this time frame is not supported.

Improving infection prevention also requires attention to non-antibiotic strategies such as preoperative screening, perioperative antisepsis, and wound care. Recent advancements, including local antibiotic prophylaxis, immunomodulatory agents, and biofilm disruption agents, offer promising avenues for enhancing infection prevention beyond traditional antibiotic-centered approaches.

Addressing the challenges of AMR and minimizing antibiotic overuse are critical for preserving treatment efficacy. This review calls for high-quality studies and tailored guidelines to optimize infection prevention practices in this specialized surgical context. By advancing our understanding and application of prophylactic strategies, we can improve patient outcomes and contribute to the global effort against AMR.

## Data Availability

All data are available upon request.

## Abbreviations

The following abbreviations are used in this manuscript:

SSI	Surgical site infection
PAP	Perioperative antibiotic prophylaxis
AMR	Antimicrobial resistance
IV	Intravenous
URTI	Upper respiratory tract infection
MRSA	Methicillin-Resistant Staphylococcus aureus
MDR	Multidrug resistance
ESBL-E	Extended-spectrum beta-lactamase-producing Enterobacterales
ASHP	American Society of Health-System Pharmacists
SOD	Selective oropharyngeal decontamination
CSD	Closed suction drains
RCT	Randomized controlled trial

## References

[1] Brook I. (2007). Microbiology and principles of antimicrobial therapy for head and neck infections. Infect. Dis. Clin. N. Am..

[2] Gearing P.F., Daly J.F., Tang N.S.J., Singh K., Ramakrishnan A. (2021). Risk factors for surgical site infection in free-flap reconstructive surgery for head and neck cancer: Retrospective Australian cohort study. Head Neck.

[3] Cheng H., Chen B.P., Soleas I.M., Ferko N.C., Cameron C.G., Hinoul P. (2017). Prolonged Operative Duration Increases Risk of Surgical Site Infections: A Systematic Review. Surg. Infect..

[4] Kubeček O., Paterová P., Novosadová M. (2021). Risk Factors for Infections, Antibiotic Therapy, and Its Impact on Cancer Therapy Outcomes for Patients with Solid Tumors. Life.

[5] Hou Y., Collinsworth A., Hasa F., Griffin L. (2023). Incidence and impact of surgical site infections on length of stay and cost of care for patients undergoing open procedures. Surg. Open Sci..

[6] Purba A.K.R., Setiawan D., Bathoorn E., Postma M.J., Dik J.H., Friedrich A.W. (2018). Prevention of Surgical Site Infections: A Systematic Review of Cost Analyses in the Use of Prophylactic Antibiotics. Front. Pharmacol..

[7] Mitchell R.M., Mendez E., Schmitt N.C., Bhrany A.D., Futran N.D. (2015). Antibiotic Prophylaxis in Patients Undergoing Head and Neck Free Flap Reconstruction. JAMA Otolaryngol. Head Neck Surg..

[8] Khariwala S.S., Le B., Pierce B.H., Vogel R.I., Chipman J.G. (2016). Antibiotic Use after Free Tissue Reconstruction of Head and Neck Defects: Short Course vs. Long Course. Surg. Infect. (Larchmt).

[9] Saunders S., Reese S., Lam J., Wulu J., Jalisi S., Ezzat W. (2017). Extended use of perioperative antibiotics in head and neck microvascular reconstruction. Am. J. Otolaryngol..

[10] Haidar Y.M., Tripathi P.B., Tjoa T., Walia S., Zhang L., Chen Y., Nguyen D.V., Mahboubi H., Armstrong W.B., Goddard J.A. (2018). Antibiotic prophylaxis in clean-contaminated head and neck cases with microvascular free flap reconstruction: A systematic review and meta-analysis. Head Neck.

[11] Carroll W.R., Rosenstiel D., Fix J.R., de la Torre J., Solomon J.S., Brodish B., Rosenthal E.L., Heinz T., Niwas S., Peters G.E. (2003). Three-dose vs extended-course clindamycin prophylaxis for free-flap reconstruction of the head and neck. Arch. Otolaryngol. Head Neck Surg..

[12] Vander Poorten V., Uyttebroek S., Robbins K.T., Rodrigo J.P., de Bree R., Laenen A., Saba N.F., Suarez C., Mäkitie A., Rinaldo A. (2020). Perioperative Antibiotics in Clean-Contaminated Head and Neck Surgery: A Systematic Review and Meta-Analysis. Adv. Ther..

[13] Karakida K., Aoki T., Ota Y., Yamazaki H., Otsuru M., Takahashi M., Sakamoto H., Miyasaka M. (2010). Analysis of risk factors for surgical-site infections in 276 oral cancer surgeries with microvascular free-flap reconstructions at a single university hospital. J. Infect. Chemother..

[14] Lahtinen S., Koivunen P., Ala-Kokko T., Kaarela O., Ohtonen P., Laurila P., Liisanantti J.H. (2018). Complications and outcome after free flap surgery for cancer of the head and neck. Br. J. Oral. Maxillofac. Surg..

[15] Septimus E.J. (2018). Antimicrobial Resistance: An Antimicrobial/Diagnostic Stewardship and Infection Prevention Approach. Med. Clin. N. Am..

[16] Goormans F., Coropciuc R., Vercruysse M., Spriet I., Willaert R., Politis C. (2022). Systemic Antibiotic Prophylaxis in Maxillofacial Trauma: A Scoping Review and Critical Appraisal. Antibiotics.

[17] Copelli C., Tewfik K., Cassano L., Pederneschi N., Catanzaro S., Manfuso A., Cocchi R. (2017). Management of free flap failure in head and neck surgery. Acta Otorhinolaryngol. Ital..

[18] Badia J.M., Casey A.L., Petrosillo N., Hudson P.M., Mitchell S.A., Crosby C. (2017). Impact of surgical site infection on healthcare costs and patient outcomes: A systematic review in six European countries. J. Hosp. Infect..

[19] Johnson J.T., Yu V.L. (1988). Antibiotic use during major head and neck surgery. Ann. Surg..

[20] Dor P., Klastersky J. (1973). Prophylactic antibiotics in oral, pharyngeal and laryngeal surgery for cancer: (a double-blind study). Laryngoscope.

[21] Piccart M., Dor P., Klastersky J. (1983). Antimicrobial prophylaxis of infections in head and neck cancer surgery. Scand. J. Infect. Dis. Suppl..

[22] Tabet J.C., Johnson J.T. (1990). Wound infection in head and neck surgery: Prophylaxis, etiology and management. J. Otolaryngol..

[23] Seagle M.B., Duberstein L.E., Gross C.W., Fletcher J.L., Mustafa A.Q. (1978). Efficacy of cefazolin as a prophylactic antibiotic in head and neck surgery. Otolaryngology.

[24] Mandell-Brown M., Johnson J.T., Wagner R.L. (1984). Cost-effectiveness of prophylactic antibiotics in head and neck surgery. Otolaryngol. Head Neck Surg..

[25] Becker G.D., Parell G.J. (1979). Cefazolin prophylaxis in head and neck cancer surgery. Ann. Otol. Rhinol. Laryngol..

[26] Ogihara H., Takeuchi K., Majima Y. (2009). Risk factors of postoperative infection in head and neck surgery. Auris Nasus Larynx.

[27] Chiesa-Estomba C.M., Lechien J.R., Fakhry N., Melkane A., Calvo-Henriquez C., de Siati D., Gonzalez-Garcia J.A., Fagan J.J., Ayad T. (2019). Systematic review of international guidelines for perioperative antibiotic prophylaxis in Head & Neck Surgery. A YO-IFOS Head & Neck Study Group Position Paper. Head Neck.

[28] Minton N.P. (2003). Clostridia in cancer therapy. Nat. Rev. Microbiol..

[29] Lee D.H., Kim S.Y., Nam S.Y., Choi S.H., Choi J.W., Roh J.L. (2011). Risk factors of surgical site infection in patients undergoing major oncological surgery for head and neck cancer. Oral Oncol..

[30] Edmiston C.E., McBain A.J., Roberts C., Leaper D. (2015). Clinical and microbiological aspects of biofilm-associated surgical site infections. Adv. Exp. Med. Biol..

[31] Shen A.Y., Lonie S., Lim K., Farthing H., Hunter-Smith D.J., Rozen W.M. (2021). Free Flap Monitoring, Salvage, and Failure Timing: A Systematic Review. J. Reconstr. Microsurg..

[32] Kamizono K., Sakuraba M., Nagamatsu S., Miyamoto S., Hayashi R. (2014). Statistical analysis of surgical site infection after head and neck reconstructive surgery. Ann. Surg. Oncol..

[33] Foster A.L., Moriarty T.F., Zalavras C., Morgenstern M., Jaiprakash A., Crawford R., Burch M.A., Boot W., Tetsworth K., Miclau T. (2021). The influence of biomechanical stability on bone healing and fracture-related infection: The legacy of Stephan Perren. Injury.

[34] Penel N., Fournier C., Lefebvre D., Lefebvre J.L. (2005). Multivariate analysis of risk factors for wound infection in head and neck squamous cell carcinoma surgery with opening of mucosa. Study of 260 surgical procedures. Oral Oncol..

[35] Tibbs M.K. (1997). Wound healing following radiation therapy: A review. Radiother. Oncol..

[36] Anderson K., Hamm R.L. (2012). Factors That Impair Wound Healing. J. Am. Coll. Clin. Wound Spec..

[37] Sieggreen M.Y. (1987). Healing of physical wounds. Nurs. Clin. N. Am..

[38] Martins Lopes M.S., Machado L.M., Ismael Amaral Silva P.A., Tome Uchiyama A.A., Yen C.T., Ricardo E.D., Mutao T.S., Pimenta J.R., Shimba D.S., Hanriot R.M. (2020). Antibiotics, cancer risk and oncologic treatment efficacy: A practical review of the literature. Ecancermedicalscience.

[39] Chiu T.H., Tsao C.K., Chang S.N., Lin J.W., Hwang J.J. (2021). Clinical consequences of head and neck free-flap reconstructions in the DM population. Sci. Rep..

[40] Cheadle W.G. (2006). Risk factors for surgical site infection. Surg. Infect..

[41] Wang Y., Wang M., Hou L., Xiang F., Zhao X., Qian M. (2023). Incidence and risk factors of surgical site infection in patients with head and neck cancer: A meta-analysis. Head Neck.

[42] Carniol E.T., Marchiano E., Brady J.S., Merchant A.M., Eloy J.A., Baredes S., Park R.C. (2017). Head and neck microvascular free flap reconstruction: An analysis of unplanned readmissions. Laryngoscope.

[43] Oppelaar M.C., Zijtveld C., Kuipers S., Ten Oever J., Honings J., Weijs W., Wertheim H.F.L. (2019). Evaluation of Prolonged vs Short Courses of Antibiotic Prophylaxis Following Ear, Nose, Throat, and Oral and Maxillofacial Surgery: A Systematic Review and Meta-analysis. JAMA Otolaryngol. Head Neck Surg..

[44] Leekha S., Terrell C.L., Edson R.S. (2011). General principles of antimicrobial therapy. Mayo Clin. Proc..

[45] Rubin J., Johnson J.T., Wagner R.L., Yu V.L. (1988). Bacteriologic analysis of wound infection following major head and neck surgery. Arch. Otolaryngol. Head Neck Surg..

[46] Finegold S.M. (1995). Anaerobic infections in humans: An overview. Anaerobe.

[47] van Mierlo A., Vaassen L., van Mens S., Kessler P. (2023). Deep Abscess Formation After Head and Neck Free Flap Reconstruction: A Critical Appraisal of Current Guidelines for Prophylactic Antibiotics in Head and Neck Surgery. J. Craniofacial Surg. Open.

[48] Rao S.V., Simon P., Saldanha E., Boloor R., Jakribettu R.P., Baliga M.S. (2024). Surgical Site Infection in Head and Neck Cancer Patients: Observations from A Tertiary Care Hospital. S. Asian J. Cancer.

[49] Onsea J., Depypere M., Govaert G., Kuehl R., Vandendriessche T., Morgenstern M., McNally M., Trampuz A., Metsemakers W.J. (2018). Accuracy of Tissue and Sonication Fluid Sampling for the Diagnosis of Fracture-Related Infection: A Systematic Review and Critical Appraisal. J. Bone Jt. Infect..

[50] Govaert G.A.M., Kuehl R., Atkins B.L., Trampuz A., Morgenstern M., Obremskey W.T., Verhofstad M.H.J., McNally M.A., Metsemakers W.J. (2020). Diagnosing Fracture-Related Infection: Current Concepts and Recommendations. J. Orthop. Trauma..

[51] Mangram A.J., Horan T.C., Pearson M.L., Silver L.C., Jarvis W.R. (1999). Guideline for Prevention of Surgical Site Infection, 1999. Centers for Disease Control and Prevention (CDC) Hospital Infection Control Practices Advisory Committee. Am. J. Infect. Control..

[52] Iocca O., Copelli C., Ramieri G., Zocchi J., Savo M., Di Maio P. (2022). Antibiotic prophylaxis in head and neck cancer surgery: Systematic review and Bayesian network meta-analysis. Head Neck.

[53] Murphy J., Isaiah A., Dyalram D., Lubek J.E. (2017). Surgical Site Infections in Patients Receiving Osteomyocutaneous Free Flaps to the Head and Neck. Does Choice of Antibiotic Prophylaxis Matter?. J. Oral. Maxillofac. Surg..

[54] Durand M.L., Yarlagadda B.B., Rich D.L., Lin D.T., Emerick K.S., Rocco J.W., Deschler D.G. (2015). The time course and microbiology of surgical site infections after head and neck free flap surgery. Laryngoscope.

[55] Johnson J.T., Wagner R.L., Schuller D.E., Gluckman J., Suen J.Y., Snyderman N.L. (1992). Prophylactic antibiotics for head and neck surgery with flap reconstruction. Arch. Otolaryngol. Head Neck Surg..

[56] White B.P., Siegrist E.A. (2021). Increasing clindamycin resistance in group A streptococcus. Lancet Infect. Dis..

[57] Bratzler D.W., Dellinger E.P., Olsen K.M., Perl T.M., Auwaerter P.G., Bolon M.K., Fish D.N., Napolitano L.M., Sawyer R.G., Slain D. (2013). Clinical practice guidelines for antimicrobial prophylaxis in surgery. Surg. Infect..

[58] Robbins K.T., Byers R.M., Cole R., Fainstein V., Guillamondegui O.M., Schantz S.P., Weber R.S., Wolf P., Goepfert H. (1988). Wound prophylaxis with metronidazole in head and neck surgical oncology. Laryngoscope.

[59] European Centre for Disease Prevention and Control Guideline on Peri-Operative Antimicrobial Prophylaxis. https://www.ecdc.europa.eu/en.

[60] NICE Guideline on Surgical Site Infections: Prevention and Treatment. https://www.nice.org.uk/guidance/ng125.

[61] Seidelman J.L., Mantyh C.R., Anderson D.J. (2023). Surgical Site Infection Prevention: A Review. JAMA.

[62] Baseel D., Kim J., Mohammed S., Lowe A., Siddiqi J. (2022). The Ideal Time to Administer Pre-operative Antibiotics: Current and Future Practices. Cureus.

[63] Vila P.M., Zenga J., Jackson R.S. (2017). Antibiotic Prophylaxis in Clean-Contaminated Head and Neck Surgery: A Systematic Review and Meta-analysis. Otolaryngol. Head Neck Surg..

[64] Balamohan S.M., Sawhney R., Lang D.M., Cherabuddi K., Varadarajan V.V., Bernard S.H., Mackinnon L.M., Boyce B.J., Antonelli P.J., Efron P.A. (2019). Prophylactic antibiotics in head and neck free flap surgery: A novel protocol put to the test. Am. J. Otolaryngol..

[65] Cohen L.E., Finnerty B.M., Golas A.R., Ketner J.J., Weinstein A., Boyko T., Rohde C.H., Kutler D., Spector J.A. (2016). Perioperative Antibiotics in the Setting of Oropharyngeal Reconstruction: Less Is More. Ann. Plast. Surg..

[66] Bartella A.K., Kamal M., Teichmann J., Kloss-Brandstätter A., Steiner T., Hölzle F., Lethaus B. (2017). Prospective comparison of perioperative antibiotic management protocols in oncological head and neck surgery. J. Craniomaxillofac Surg..

[67] Zimmerli W., Waldvogel F.A., Vaudaux P., Nydegger U.E. (1982). Pathogenesis of foreign body infection: Description and characteristics of an animal model. J. Infect. Dis..

[68] Obremskey W.T., Metsemakers W.J., Schlatterer D.R., Tetsworth K., Egol K., Kates S., McNally M. (2020). Musculoskeletal Infection in Orthopaedic Trauma: Assessment of the 2018 International Consensus Meeting on Musculoskeletal Infection. J. Bone Jt. Surg. Am..

[69] Gajic I., Kabic J., Kekic D., Jovicevic M., Milenkovic M., Mitic Culafic D., Trudic A., Ranin L., Opavski N. (2022). Antimicrobial Susceptibility Testing: A Comprehensive Review of Currently Used Methods. Antibiotics.

[70] Laxminarayan R., Duse A., Wattal C., Zaidi A.K., Wertheim H.F., Sumpradit N., Vlieghe E., Hara G.L., Gould I.M., Goossens H. (2013). Antibiotic resistance-the need for global solutions. Lancet Infect. Dis..

[71] Centers for Disease Control and Prevention Antibiotic Resistance Threats in the United States. https://www.cdc.gov/antimicrobial-resistance/media/pdfs/2019-ar-threats-report-508.pdf?CDC_AAref_Val=https://www.cdc.gov/drugresistance/pdf/threats-report/2019-ar-threats-report-508.pdf.

[72] Rahman M.M., Alam Tumpa M.A., Zehravi M., Sarker M.T., Yamin M., Islam M.R., Harun-Or-Rashid M., Ahmed M., Ramproshad S., Mondal B. (2022). An Overview of Antimicrobial Stewardship Optimization: The Use of Antibiotics in Humans and Animals to Prevent Resistance. Antibiotics.

[73] Schuts E.C., Hulscher M., Mouton J.W., Verduin C.M., Stuart J., Overdiek H., van der Linden P.D., Natsch S., Hertogh C., Wolfs T.F.W. (2016). Current evidence on hospital antimicrobial stewardship objectives: A systematic review and meta-analysis. Lancet Infect. Dis..

[74] Bhalodi A.A., van Engelen T.S.R., Virk H.S., Wiersinga W.J. (2019). Impact of antimicrobial therapy on the gut microbiome. J. Antimicrob. Chemother..

[75] Branch-Elliman W., O’Brien W., Strymish J., Itani K., Wyatt C., Gupta K. (2019). Association of Duration and Type of Surgical Prophylaxis With Antimicrobial-Associated Adverse Events. JAMA Surg..

[76] Blumenthal K.G., Peter J.G., Trubiano J.A., Phillips E.J. (2019). Antibiotic allergy. Lancet.

[77] Pascale A., Marchesi N., Marelli C., Coppola A., Luzi L., Govoni S., Giustina A., Gazzaruso C. (2018). Microbiota and metabolic diseases. Endocrine.

[78] Kraemer S.A., Ramachandran A., Perron G.G. (2019). Antibiotic Pollution in the Environment: From Microbial Ecology to Public Policy. Microorganisms.

[79] Shui L., Yang X., Li J., Yi C., Sun Q., Zhu H. (2019). Gut Microbiome as a Potential Factor for Modulating Resistance to Cancer Immunotherapy. Front. Immunol..

[80] Reed J.P., Devkota S., Figlin R.A. (2019). Gut microbiome, antibiotic use, and immunotherapy responsiveness in cancer. Ann. Transl. Med..

[81] Ma W., Mao Q., Xia W., Dong G., Yu C., Jiang F. (2019). Gut Microbiota Shapes the Efficiency of Cancer Therapy. Front. Microbiol..

[82] Tinsley N., Zhou C., Tan G., Rack S., Lorigan P., Blackhall F., Krebs M., Carter L., Thistlethwaite F., Graham D. (2020). Cumulative Antibiotic Use Significantly Decreases Efficacy of Checkpoint Inhibitors in Patients with Advanced Cancer. Oncologist.

[83] Galli G., Triulzi T., Proto C., Signorelli D., Imbimbo M., Poggi M., Fucà G., Ganzinelli M., Vitali M., Palmieri D. (2019). Association between antibiotic-immunotherapy exposure ratio and outcome in metastatic non small cell lung cancer. Lung Cancer.

[84] Nenclares P., Bhide S.A., Sandoval-Insausti H., Pialat P., Gunn L., Melcher A., Newbold K., Nutting C.M., Harrington K.J. (2020). Impact of antibiotic use during curative treatment of locally advanced head and neck cancers with chemotherapy and radiotherapy. Eur. J. Cancer.

[85] Liu Z., Dumville J.C., Norman G., Westby M.J., Blazeby J., McFarlane E., Welton N.J., O’Connor L., Cawthorne J., George R.P. (2018). Intraoperative interventions for preventing surgical site infection: An overview of Cochrane Reviews. Cochrane Database Syst. Rev..

[86] Lanzetti J., Finotti F., Savarino M., Gassino G., Dell’Acqua A., Erovigni F.M. (2023). Management of Oral Hygiene in Head-Neck Cancer Patients Undergoing Oncological Surgery and Radiotherapy: A Systematic Review. Dent. J..

[87] Price R., MacLennan G., Glen J. (2014). Selective digestive or oropharyngeal decontamination and topical oropharyngeal chlorhexidine for prevention of death in general intensive care: Systematic review and network meta-analysis. BMJ.

[88] Bohorquez D., Pena S., Weed D., Ma R., Arnold D.J. (2022). Effect of Drain Output on the Timing of Closed Suction Drain (CSD) Removal After Head and Neck Surgery. Cureus.

[89] Huiras P., Logan J.K., Papadopoulos S., Whitney D. (2012). Local antimicrobial administration for prophylaxis of surgical site infections. Pharmacotherapy.

[90] Metsemakers W.J., Fragomen A.T., Moriarty T.F., Morgenstern M., Egol K.A., Zalavras C., Obremskey W.T., Raschke M., McNally M.A. (2020). Evidence-Based Recommendations for Local Antimicrobial Strategies and Dead Space Management in Fracture-Related Infection. J. Orthop. Trauma..

[91] Pirofski L.A., Casadevall A. (2006). Immunomodulators as an antimicrobial tool. Curr. Opin. Microbiol..

[92] Mahmud F., Roy R., Mohamed M.F., Aboonabi A., Moric M., Ghoreishi K., Bayat M., Kuzel T.M., Reiser J., Shafikhani S.H. (2022). Therapeutic evaluation of immunomodulators in reducing surgical wound infection. Faseb J..

[93] Coppola G.A., Onsea J., Moriarty T.F., Nehrbass D., Constant C., Zeiter S., Aktan M.K., Braem A., Van der Eycken E.V., Steenackers H.P. (2021). An Improved 2-Aminoimidazole Based Anti-Biofilm Coating for Orthopedic Implants: Activity, Stability, and in vivo Biocompatibility. Front. Microbiol..

[94] Mirani Z.A., Fatima A., Urooj S., Aziz M., Khan M.N., Abbas T. (2018). Relationship of cell surface hydrophobicity with biofilm formation and growth rate: A study on Pseudomonas aeruginosa, Staphylococcus aureus, and Escherichia coli. Iran. J. Basic. Med. Sci..

[95] Liu A., Fong A., Becket E., Yuan J., Tamae C., Medrano L., Maiz M., Wahba C., Lee C., Lee K. (2011). Selective advantage of resistant strains at trace levels of antibiotics: A simple and ultrasensitive color test for detection of antibiotics and genotoxic agents. Antimicrob. Agents Chemother..

[96] Stanton I.C., Murray A.K., Zhang L., Snape J., Gaze W.H. (2020). Evolution of antibiotic resistance at low antibiotic concentrations including selection below the minimal selective concentration. Commun. Biol..

[97] Verderosa A.D., Totsika M., Fairfull-Smith K.E. (2019). Bacterial Biofilm Eradication Agents: A Current Review. Front. Chem..

